# Combining Zirconia and Titanium Suboxides by Vat Photopolymerization

**DOI:** 10.3390/ma14092394

**Published:** 2021-05-04

**Authors:** Eric Schwarzer-Fischer, Anne Günther, Sven Roszeitis, Tassilo Moritz

**Affiliations:** Processes and Components, Fraunhofer Institute for Ceramic Technologies and Systems, 01277 Dresden, Germany; eric.schwarzer-fischer@ikts.fraunhofer.de (E.S.-F.); anne.guenther@ikts.fraunhofer.de (A.G.); sven.roszeitis@ikts.fraunhofer.de (S.R.)

**Keywords:** additive manufacturing, vat photopolymerization (VPP), hybridization, multi-functional components, zirconia, titanium suboxides, Magnéli phases

## Abstract

A recently developed multi-ceramic additive manufacturing process (multi-CAMP) and an appropriate device offer a multi-material approach by vat photopolymerization (VPP) of multi-functionalized ceramic components. However, this process is limited to ceramic powders with a certain translucency for visible light. Electrically conductive ceramic powders are therefore ruled out because of their light-absorbing behavior and dark color. The goal of the collaborative work described in the article was to develop a material combination for this multi-material approach of the additive vat photopolymerization method which allows for combining electrical conductivity and electrical insulation plus high mechanical strength in co-sintered ceramic components. As conductive component titanium suboxides are chosen, whereas zirconia forms the mechanically stable and insulation part. Since titanium suboxides cannot be used for vat photopolymerization due to their light-absorbing behavior, titania is used instead. After additive manufacturing, the two-component parts are co-sintered in a reducing atmosphere to transform the titania into its suboxides and, thus, attaining the desired property combination. The article describes the challenges of the co-processing of both materials due to the complex optical properties of titania. Furthermore, the article shows successfully co-sintered testing parts of the material combination of zirconia/titanium suboxide which are made by assembling single-material VPP components in the green state and subsequent common thermal treatment. The results of microstructural and interface investigations such as electrical measurements are discussed.

## 1. Introduction

Additive manufacturing (AM) offers countless degrees of freedom in the geometric design of components. If, in addition, these manufacturing methods can be used for combining materials with different properties, then further degrees of freedom are added in the multi-functionalization of these components. However, most of the AM devices which currently exist on the market are only applicable to single material applications. In the field of ceramic components, the situation is much more complicated because AM of ceramics, as is known for all other conventional ceramic shaping techniques, means powder technology. After the shaping process, regardless of whether additively or conventionally manufactured, so-called ceramic green parts must be debound and sintered to get their final desired properties. These thermal treatment steps complicate the processing chain and can be seen as the main reason why AM of ceramics in terms of market penetration is behind such methods for metals or polymers. This issue is becoming even more complex when two or more ceramic materials are combined to obtain components with multi-functional properties, like differing colors, porous and dense structures, electrical conductivity and insulation or different mechanical properties. Co-shaping of different components is very attractive for the production of multi-functional parts because additional expensive joining steps can be neglected [1]. However, after co-shaping, the green parts must be co-debound and co-sintered as well [2]. The selection of a suitable material pairing is a crucial factor for a successful co-sintering process, as chemical and physical properties cannot simply be changed by process adjustments [3]. At first, the materials have to match chemically in order to allow bonding on an atomic scale [4]. Additionally, since they are joined by co-sintering, they must be compatible in sintering and thermal expansion behavior [5,6]. On the technological scale, the shrinkage of the feedstocks has to be adjusted while maintaining their processability [7].

The conventional stereolithography (SLA) process, also called vat photopolymerization (VPP), is one of the best-known processes in AM of ceramic components. Halloran et al. [8] first developed SLA technology applied to ceramics. It is one of the major processes for polymers and applications for ceramics, including alumina [9], silica [10], silicon nitride [11,12] and zirconia-toughened alumina [11]. As the basis of this process, photopo-lymerizable suspensions filled with ceramic particles are cured by a UV laser. In [13], two different types of VPP processes are described which are commercially available now: (1) a top-down system with a scanning laser on top and (2) a bottom-up system with digital light projection. The top-down VPP process uses a low-power, highly focused UV laser beam to scan successive cross-sections of the three-dimensional object in a vat of liquid photosensitive polymer.

As the laser scans the layer, the polymer is cured, and the excess areas are left as slurry. After the platform has been lowered for the next layer, a squeegee is moved over the surface to smooth it before scanning. The platform is lowered by a distance equal to the layer thickness (25 to 50 µm), and a subsequent layer is formed on top of the previously cured layers. This process is continued until the component is built. Excess suspension is washed away from the built component. For final curing, the part is placed in a UV oven. The manufacturing process requires the formulation of a photoreactive suspension containing a photocurable resin and the ceramic powder before exposure to visible or UV light. Once polymerized, the photopolymer constitutes a composite of the interconnected polymer matrix around ceramic particles and provides the cohesion of the green body. The polymer matrix is subsequently removed by an appropriate thermal debinding step; subsequently, the sintering of the green part ensures the final properties of the ceramic pieces.

Indeed, bottom-up systems have become widely used in the past several years. A light source consisting of an LED lamp, in conjunction with a liquid crystal display panel or a deformable mirror device, exposes a whole layer of light-curable suspension from below through a transparent window or a carrier tape at once.

According to [14], a candidate for a ceramic suspension for the VPP process must fulfill several requirements:

- high particle volume loading for dense and high-quality ceramic pieces after debinding and sintering.
- minimal concentration of organics in the suspension to avoid deformation and cracking during binder removal, to reduce the sintering shrinkage and to obtain defect-free ceramic parts after sintering.
- low suspension viscosity < 5 Pa·s, to ensure satisfactory layer recoating and for shorter leveling of a freshly applied layer.
- curability of the suspension upon exposure of UV or visible light with sufficient depth and resolution.

The behavior of the resin under photopolymerization is strongly influenced by the adjunction of particles due to scattering and absorption of the incident light beam by the particles. Powder suspensions can have a very high turbidity due to radiation scattering, even if the ceramic itself is transparent to UV or visible light. This scattering-induced turbidity reduces the curing depth for a ceramic suspension. The scattering ability of a material is described in the scattering efficiency term *Q*, where *Q* is a function of the refractive index difference between the ceramic and the UV-curable solution [15]:

(1) $$Q=\beta \Delta n^{2}$$

The cure depth is inversely proportional to $\Delta n^{2}={\left(n_{ceramic}-n_{resin}\right)}^{2}$, where *n* is the refractive index. The term *β* is related to the interparticle spacing and wavelength of the radiation. For that reason, the ceramic material which is used for the VPP process should have: (1) a certain translucency for light with the wavelength applied for curing, and (2) a refractive index possibly close to the refractive index of the suspension media, i.e., the photocurable resin.

Information on AM devices suited for the manufacturing of multi-functional ceramic components are hard to find. A machine for the so-called multi-ceramic additive manufacturing process (multi-CAMP) has been developed by the Korean Institute for Materials Science (KIMS) with material combinations to obtain multi-functional ceramic components. This device uses the bottom-up approach described above. Two carrier tapes provide the light curable slurries of different ceramic powders. The tapes are moved below the building platform and the components are built layer-wise hanging top-down on the building platform by exposure to blue light with a wavelength of 405 nm from a light source situated below the tape. If a material change takes place within one component, the building platform is moved from tape A by a 180° swivel to tape B. After 90°, the platform is stopped for an intermediate cleaning step. For that purpose, a cleaning station is positioned in between both tapes. Uncured, liquid slurry can be removed for avoiding any cross-contamination between both ceramic slurries. In a research project between KIMS and Fraunhofer IKTS, Germany, a material combination for coupling electrical conductivity/electrical insulation could be developed which can be processed in the multi-CAMP device. The chances and challenges of this development work are described in this article.

A wide range of ceramics are available for the desired property combination of electrical conductivity and electrical insulation. However, ceramics with electron conductivity are light absorbing and cannot be used as initial powders for VPP processes like the multi-CAMP. For that reason, for combining electrically conductive and insulating ceramics by VPP, a detour must be taken. A material coupling for the desired property combination of zirconia and titanium suboxides was chosen. Zirconia is well known for its mechanical strength [16,17] and electrical insulation [17], whereas titania suboxides are famous for being electrical conductors, additionally showing thermoelectrical properties [18]. The chosen material combination offers several promising fields of application as electrodes for electrolytic water splitting or for the generation of atmospheric pressure plasma, for electrical heaters, sensors or for thermoelectrical generators. Titanium suboxide electrodes are already being produced commercially [19,20]. In particular, their high chemical stability, sufficiently good electrical conductivity and high abrasion resistance distinguish them from metal or graphite electrodes. Titanium suboxides are oxides that deviate from the stoichiometry of titanium dioxide. Titanium suboxides can be modified in wide ranges with respect to their electrical conductivity [21,22]. For example, titanium dioxide (TiO_2_) is electrically insulating with a resistivity of R_sp_ = 1013 Ω·cm. The Magnéli phase Ti_4_O_7_ with an R_sp_ ~ 10^−3^ Ω·cm achieves metal-like conductivity. Between the two extreme values of 1013 and ~10^−3^ Ω·cm, almost all electrical resistances can be adjusted by mixing the individual phases of the different titanium oxides. Especially in the range of Magnéli phases of Ti_n_O_2n−1_ with 4 < n < 10, it is possible to adjust the electrical resistivity of titanium suboxide ceramics from 10^−1^ to 10^−3^ Ω·cm. For this purpose, two neighboring Magnéli phases can be mixed, which then obtain a defined electrical resistivity according to the mixing ratio. The preparation of such ceramics has been described in [21,23]. Further options for resistance matching are provided by Ti-O compounds outside the range of Magnéli phases [24]. The defined adjustment of the electrical properties has been the subject of various fundamental studies in the past [21,24]. In particular, work has been carried out with single-phase titanium suboxides and calculations have been performed on the basis of solid-state physics. Their use as thermoelectric materials was also discussed, where, besides the electrical conductivity, the thermal conductivity and the Seebeck coefficient are important [21,25]. The co-sintering of different Magnéli phases with each other has already been described in [25].

However, combining titanium suboxides with zirconia has not yet been reported, neither by conventional shaping nor by AM. Combining both materials by VPP requires one to solve some specific problems. Since titania suboxides exhibit UV–Vis diffuse reflectance spectra of the nanoparticle absorption beyond 325 nm, and an absorption band that covers the visible light wavelengths and extends into the near-infrared region [26], for the building process by multi-CAMP, pure TiO_2_ is used which is combined with a ZrO_2_ component. Titania is a white and translucent powder, but after the VPP building process, the TiO_2_ must be converted into the suboxide phase during the sintering step reduce the lattice oxygen and to get the desired electrical and thermoelectric properties of the final component.

A further limit for the VPP process is that TiO_2_ shows an absorption edge for UV light very close to 405 nm which is used by the multi-CAMP machine. For that purpose, in the AM process used in this article, a DLP module emitting light with a wavelength between 452 and 465 nm was used. Furthermore, the TiO_2_ particles scatter the light due to a very high refraction index of the material (2.5…3.0 depending on the crystal modification and a wavelength below 500 nm).

For the subsequent conversion of TiO_2_ into TiO_x_, a special co-sintering process must be developed for sufficient densification of both components.

This article describes the development of ZrO_2_/TiO_x_ material compounds as a material combination suited for the multi-material approach of the additive vat photopolymerization method, which allows for combining electrical conductivity and electrical insulation plus high mechanical strength in co-sintered ceramic components. It gives detailed information on the light-curable slurry development of the initial ZrO_2_ and TiO_2_ powders, the manufacturing of testing parts for the material compound by using a single material VPP device and the co-sintering process of the material compound which is not only used for densification of both ceramic components, but, moreover, transforms the TiO_2_ into conductive titanium suboxides. Finally, the results of microstructural investigations of the compound interface and electrical properties of the attained titanium suboxide mixture are shown and discussed.

## 2. Materials and Methods

### 2.1. Ceramic Slurry Development

At first, the base material for “printing” must be developed by tailoring the raw materials—ceramic powders, dispersing agents, monomers and initiators—to attain a highly particle-filled stable slurry with moderate flow and curing properties to suit the process requirements. The development started with the selection of a titania (KRONOS 1002 from Kronos International, Inc. Leverkusen, Germany) and a zirconia (partially stabilized with 3 mol.-% yttria; TZ‑3YS‑E from TOSOH, Tosoh Bioscience A.G., Griesheim, Germany) powder, which were characterized concerning their morphological properties. The zirconia powder TZ-3YS-E from TOSOH is one of the most commonly used powders in the field of advanced ceramic components. The initial powder for the ceramic counterpart, the titania powder KRONOS 1002, was chosen for co-sintering reasons. This powder should have a quite similar particle size distribution to attain similar green densities of both components and comparable sintering behavior. The particle size and the specific surface of both powder materials were determined by laser diffraction (Mastersizer 2000, Malvern Panalytical Ltd., Kassel, Germany) and nitrogen absorption by the BET method (ASAP2020 Micromeritics GmbH, Aachen, Germany).

Novel ceramic slurries were developed for these materials, which can be used in lithography-based additive manufacturing processes with a light source of a wavelength of 458 nm.

Based on these components, photoreactive zirconia and titania slurries with a powder content up to 51 vol.% were prepared and characterized. The ceramic particles were dispersed in a diluent together with the dispersant, a mixture of various monomers and a photoinitiator which fits the wavelength was used for the printing process. Various mo-nomer compositions were tested, two of them were characterized in more detail as resin composition (rc) for slurry development (Table 1).

Comparative investigations for the two ceramic powders were carried out with the two given compositions by using a ceramic solids content of 46 vol.%, a plasticizer (PEG 400 polyethylene glycol, Sigma-Aldrich by Merck KGaA, Darmstadt, Germany) content of 30 wt.% related to photoreactive organics (acrylates) and an initiator (99% Camphor quinone (CQ) with co-initiator ethyl 4-dimethylaminobenzoate, both from Alfa Aesar, Landau, Germany) content of 1% in relation to the resin quantity. Final printing tests and the investigation of the sinter shrinkage were done by using slurries with a solid content up to 50 vol.%.

A planetary centrifugal high-speed vacuum mixer (Thinky ARV310, C3-Prozesstech und Analysentechnik, Haar, Germany) was used for the stepwise (three times for 5 min at 2000 rpm) preparation of the slurries. After slurry preparation, different investigations were done concerning optimal processing parameters for VPP, like coating speed and energy dose for exposure. Furthermore, the slurries were characterized regarding their rheological properties (Modular Compact Rheometer MCR302 with a cone/plate measurement system, Anton Paar, Graz, Austria) and curing behavior. Viscoelastic behavior (low dynamic viscosity at high shear rates) in a low viscosity range (below 30 Pa·s at a shear rate of 10 s^−1^) is optimal for use in VPP, due to the rotational slurry coating mechanism of the process. Therefore, the flow behavior, especially the dynamic viscosity, was measured by stressing with shear rates in a range of 0.1 to 1000 s^−1^. The curing by light exposure was determined by measuring the curing depth of a specimen (approx. 1 mL) depending on the energy dose (smart LED with 458 nm wavelength), a value resulting from the irradiation intensity (adjusted by a photometer) with time. Based on these, process parameters for VPP printing were derived and necessary optimizations regarding flow and cross-linking behavior were carried out by adjusting the compositions.

### 2.2. VPP of Testing Samples

For manufacturing of the testing samples, the VPP technology (which is also known as lithography-based ceramic manufacturing (LCM) [27]) based on the selective initiation of photopolymerization in a highly particle-filled slurry (typical particle sizes: between 0.04 and 5 µm; typical ceramic content: 35–55 vol.%) by irradiation with blue light (wavelength: 452–465 nm) was applied. First, test samples were manufactured by means of the LCM technology, especially with the CeraFab 7500 printing device (Lithoz GmbH, Vienna, Austria). Within this process, a layer of the highly particle-filled slurry is applied by the rotation of a vat in combination with a static wiper blade. The bottom of the vat is transparent so the light source can locally expose the slurry from below. Using a dedicated optical system, the projected image is generated via a digital micromirror device (DMD) at a high resolution (min. 1920 × 1080 pixels), to realize a 40 µm pixel size in the x–y plane and a minimum wall thickness of as low as 100 µm in the sintered parts. The achievable tolerances are in the range of 40–100 µm (known roughness values are R_a_ value of 0.4 to 2 µm).

By using this technology, first, single test components like cubes (edge size of 5 mm), bars (square cross-section of 1–5 mm edge size and length of 40 mm) and special demonstrators with different geometric features were manufactured to validate the printing for both ceramic slurries. After printing, the slurry remaining on the part’s surface was removed as much as possible with a specific cleaning solution in combination with compressed air. The next step in the process chain was a comprehensive characterization including visual or microscopic sample inspection and size and weight measurements.

### 2.3. Thermal Treatment

An essential step in the processing chain is the thermal treatment, including the complete removal of organics by debinding and, afterwards, sintering. Debinding is a very critical step in the process chain because it may cause cracks and defects due to excessive outgassing if the process is not carried out correctly. To avoid this, thermogravimetric analyses (TGAs) can be used to develop a debinding strategy adapted to the materials. Within the TGA, the mass loss of organics depending on the temperature is quantified. Based on these data, a defined temperature profile with heating rates and dwell times can be developed and adjusted. TGAs were carried out by using the thermal analyzer STA 429 apparatus (NETZSCH-Gerätebau GmbH, Selb, Germany) for a few selected systems that fulfill the specified printing requirements of the VPP process. For this purpose, small fragments of printed test specimens of the different material systems were thermally treated both in air and in a nitrogen atmosphere at a heating rate of 1 K/min up to 600 °C. Afterwards, a heating profile for debinding was developed and checked by using a changeable atmosphere furnace (CAF; MUT Advanced Heating GmbH, Jena, Germany) for debinding the samples smoothly under a nitrogen atmosphere of up to 600 °C.

In the present case, sintering is also a delicate matter, since, on the one hand, a defined TiO_x_ phase must be obtained for a moderate electrical conductivity and, on the other hand, the zirconia must have an appropriate density (>99%) for a high strength (>700 MPa).

Co-sintering of ZrO_2_ with TiO_2_ was determined by the desired conversion of the TiO_2_ to TiO_x_. A maximum sintering temperature of the materials was set at 1400 °C. Due to the described reduction, sintering must be carried out in reducing gases and a vacuum and must be thermodynamically safe so that the desired Magnéli phases of the TiO_x_ are achieved. The sintering regime is shown in Table 2 and was carried out in forming gas of 95% nitrogen/5% hydrogen (5 L/min N_2_ + flushing 0.25 L/min H_2_) as follows:

The individual regime stages result from specific conversion steps of the TiO_x_. The conversion from the TiO_2_ modification anatase to rutile takes place at approx. 700 °C, which is the reason why the heating rate must be reduced. With the start of sintering of the TiO_2_ or TiO_x_ at 800–900 °C, a lower heating rate is necessary for homogeneous shrinkage with controlled diffusion processes. The active reduction phase begins at 1000 °C and ends at approx. 1200 °C. Especially in the final phase, the reduction could take place homogeneously during a long holding time of 1020 min in the entire TiO_x_ substrate, accompanied by gas formation. The rest of the sintering takes place in the range 1200–1400 °C in a vacuum to prevent further reduction. The holding time at the maximum temperature is 120 min and is carried out under N_2_. Sintering and shrinkage of TiO_x_ ends at 1300–1400 °C depending on the Magnéli phase to be achieved, and for ZrO_2_ at 1400 °C. For complete densification of TiO_x_, a sintering temperature of 1450 °C is also tested. Cooling takes place in forming gas to room temperature to avoid renewed oxidation of the TiO_x_ phase.

### 2.4. Sinter Shrinkage Adjustment

**Figure 1:**
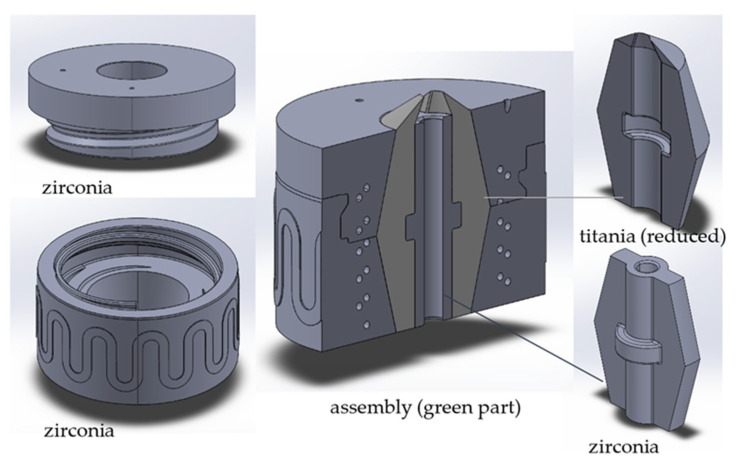
Custom-fit core–shell assembly consisting of four single material components printed by VPP.

For the main goal, realizing a two-material component by means of the multi-CAMP device, sintering shrinkage adjustment was necessary for the TiO_x_–ZrO_2_ system. At first, the sintering shrinkage of printed titania and zirconia samples with different solid contents was analyzed to obtain the relationship between total shrinkage and solids content under the same sintering conditions for both materials. For this purpose, square rods (dimensions of 5 × 5 × 25 mm^3^) were printed with the long side (25 mm) oriented in x, y and z directions. The dimensions of the samples in the green and sintered state were measured by using a digital caliper (accuracy of ±30 µm, Mitutoyo Deutschland GmbH, Neuss, Germany). For a first validation, a type of core–shell system (see Figure 1) was developed to combine separately printed samples of titania and zirconia with a corresponding solid content in the green state. Thus, a custom-fit test section was created including the two materials in one component, that could be thermally co-processed within one step. The assembled green components were then thermally treated, and the result was characterized.

### 2.5. Characterization

Based on the dimension changes of the test samples (cubes and bars) between the as-printed and sintered state, which were measured by a digital caliper before and after sintering, due to sinter shrinkage, the printing correction factors were calculated. The printing correction factors for all dimensions were the oversize which must be used during printing to achieve the required and correct dimensions of a part after sintering. The values for the respective spatial directions resulted from the ratio of the green size of a part to its sintered dimensions. For both materials and the different solid contents, a minimum of 10 printed parts for all orientations were measured. Furthermore, the density of the components was measured by hydrostatic weighing.

**Figure 2:**
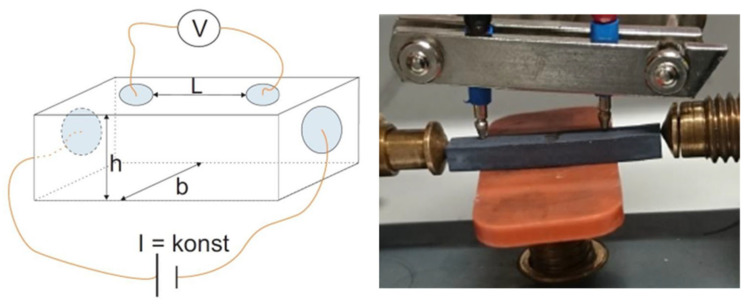
Four-point method for measuring the electrical conductivity with change of polarization.

Field Emission Scanning Electron Microscope FESEM (Gemini 982, Carl Zeiss Microscopy GmbH, Oberkochen, Germany; samples sputtered with carbon) and X-ray diffraction XRD (D8 Advance, Bruker Corporation, Billerica, MS, USA) investigations were carried out on cross-sections of sintered testing parts. The interface between zirconia and titanium suboxide was investigated by Energy-dispersive X-ray spectroscopy EDX measurements. The electrical conductivity was measured via a four-point method (Figure 2) with changes of polarization to eliminate the influence of the thermoelectric Seebeck effect of the titanium suboxides. The current (I) remained constant and the corresponding resistance could be measured by adjusting the voltage.

TiO_x_ rods with different cross-sectional edge lengths (h and b; in our case h = b = 1 mm, 2 mm or 3 mm) sintered by using the developed profile (1400 °C) were clamped between the two lateral contacts for this purpose. By placing the two upper points on the machined edge, an electrical voltage was generated within the rod. The resulting resistance was then determined and converted into an electrical conductivity depending on the sample properties (dimensions and density).

## 3. Results

### 3.1. Feedstock Development and Validation by Printing

**Figure 3:**
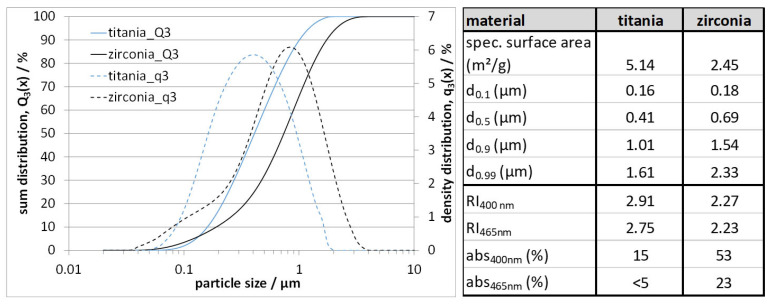
Particle size distribution (sum distribution: Q_3_(x) and density distribution: q_3_(x)) of titania and zirconia raw material (left); table comparing the specific characteristic sizes and the specific surface area, where “d” denotes the diameter at a given permeate of an analytical mesh which has been formerly used for sieve analysis, i.e., 0.1 = 10% flux through a mesh, 0.9 = 90% flux through a mesh.

Figure 3 shows the particle size of both initial powders. Additionally, the specific characteristic sizes, the specific surface area and optical properties—refractive index (RI) and absorption (abs) depending on the wavelength [28,29,30]—are compared in the given table. A verification of the presented property values was not done.

Both materials have a comparable particle size in the submicron range, suitable for the used lithography processes. The titania raw material is slightly finer with a mean particle size of 0.41 µm in contrast to the zirconia at 0.69 µm. Both materials must be deagglomerated as much as possible during slurry preparation to get the best material properties. One striking difference between the two materials can be found in the refractive index, which is 2.75–2.91 for titania, significantly higher compared to zirconia, at 2.23–2.27. Since the cure depth in a photopolymeric slurry system is inversely proportional to the refractive index difference between the ceramic and the UV-curable solution, these values could be decisive for achieving moderate layer cure depths for both materials as a requirement for the printing process. The absorption, even if it is quite high for zirconia at approx. 53%, can be neglected here, as the applicability of such systems in stereolithography printing with moderate layer thicknesses has already been proven.

**Figure 4:**
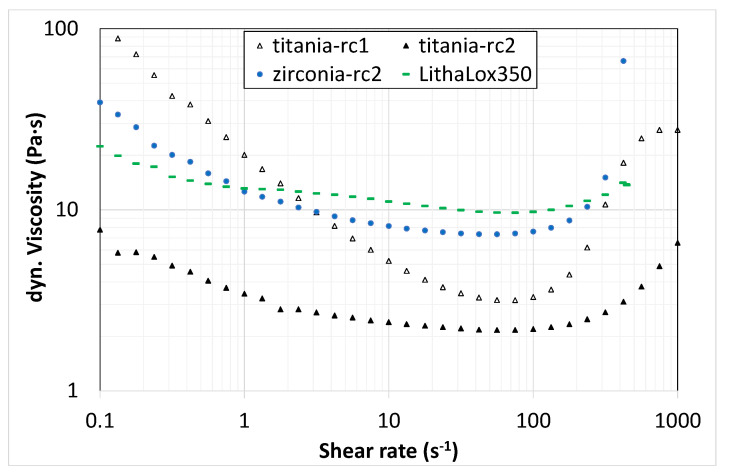
Dynamic viscosity of various titania (triangles) and zirconia (circles) suspensions compared to a commercial suspension (Lithalox 350).

**Figure 5:**
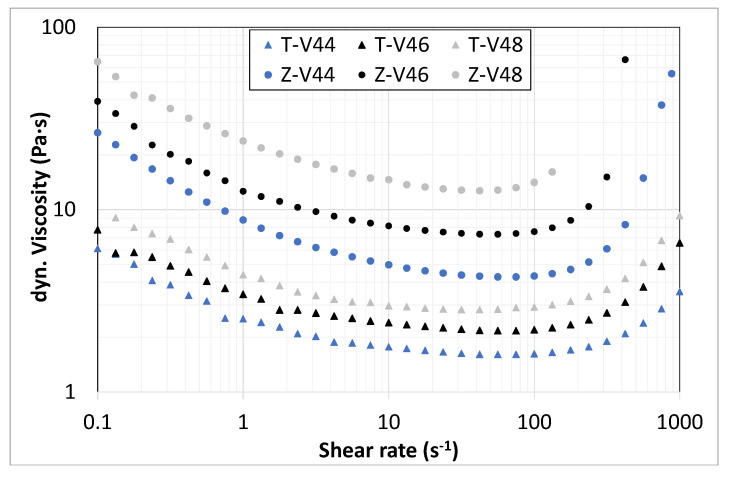
Viscosity of titania (triangles) and zirconia (circles) slurries based on rc2 depending on shear rate; solids content of 44, 46 and 48 vol.%.

During the investigation, various resin compositions (rc) were figured out for tests as a binder formulation in suspension development. Based on these components, zirconia and titania suspensions with a powder content up to 51 vol.% were prepared and characterized concerning their flow and curing behavior. In Figure 4 and Figure 5, the flow and curing behavior of different titania as well as zirconia suspensions are presented. The flow behavior was determined from the dynamic viscosity and compared with a commercially available suspension (Lithalox 350, Al_2_O_3_).

Figure 4 shows the dynamic viscosity depending on the shear rate of two titania suspensions with solid contents of 46 vol.% based on the resin mixtures rc1 and rc2, and the proportion of dispersant was kept constant (approx. 1.2 wt.% regarding particle content). Both suspensions show a typical shear thinning behavior, but they completely differ in the level of viscosity. Resin composition rc1 has a substantially higher initial viscosity of 100 Pa·s, which drops rapidly with increasing shear rate up to 100 s^−1^ (<3 Pa·s), followed by shear thickening behavior to a viscosity of up to 30 Pa·s. The suspension based on rc2 has a much lower initial viscosity of 8 Pa·s (at 0.1 s^−1^), which drops to 2 Pa·s (100 s^−1^), followed by a smooth increase up to 7 Pa·s (1000 s^1^). One possible reason for this could be the significantly higher initial viscosity of the aliphatic urethane acrylate of resin composition rc1. Nevertheless, due to the much lower viscosity of rc2, only this resin composition was used for zirconia suspensions and in this case compared for a 46 vol.% solids content. The zirconia suspension also shows a shear thinning behavior which is comparable to the less viscous titania suspension (rc2), but the level of the viscosity is even higher with an initial value of 30 Pa·s (0.1 s^−1^), decreasing to 8 Pa·s (100 s^−1^) and is followed by shear thickening back to 30 Pa·s (1000 s^−1^). Overall, the behavior almost matches the reference suspension, so similar process parameters can be used during printing with VPP.

In the next step, the influence of the solid content on the viscosity of suspensions based on resin composition rc1 (at constant dispersant content) is compared for both materials. The result is presented in Figure 5.

Characteristically, the viscosity level for both materials increases with increasing solid contents. Regardless of this, the values for the viscosity of the investigated solid contents are within the range usable for VPP.

**Figure 6:**
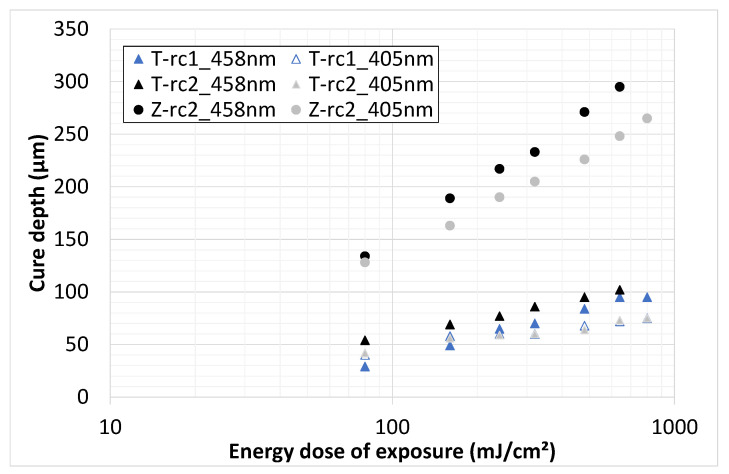
Cure depth vs (log) energy dose; titania (T) slurries based on rc1 and rc2; zirconia (Z) slurry based on rc2; particle content of 46 vol.%; 405 and 458 nm wavelength.

The results of the curing characterization are shown in the following section. For the different slurries, the depth of cure depends on various parameters such as material, solids content, resin composition, initiator content, wavelength, energy intensity and dose, for instance. Starting with a comparison of both ceramic materials (solids content of 46 vol.%) and the two resin compositions, the cure depth is compared depending on energy dose and wavelength (see Figure 6).

There is a significant difference in the cure depth between the two materials irrespective of the wavelength, whereby significantly higher (three times on average) cure depths were determined for zirconia. The reason for this is seen in the higher refractive index of the titania and not in the absorption. It is known, and has already been mentioned above, that the depth of curing depends quadratically on the refractive index difference between the ceramic material and the resins. In this case, this must necessarily have led to the major differences in cure depth. Only minor differences were found between the two resin compositions rc1 and rc2. It can be assumed that the reactivity and the optical properties are comparable. However, there are greater differences between the wavelengths. Reducing the wavelength to 405 nm led to an average decrease in cure depth of 20% for the zirconia and 40% for the titania. The cause of the reduction for ZrO_2_ could be associated with the significant increase in absorption (from 15 to 53%), because the refractive index is nearly constant. For titania, the reduction must be related to the increase in refractive index (from 2.7 to 2.9), as the absorption only increases by 10%.

**Figure 7:**
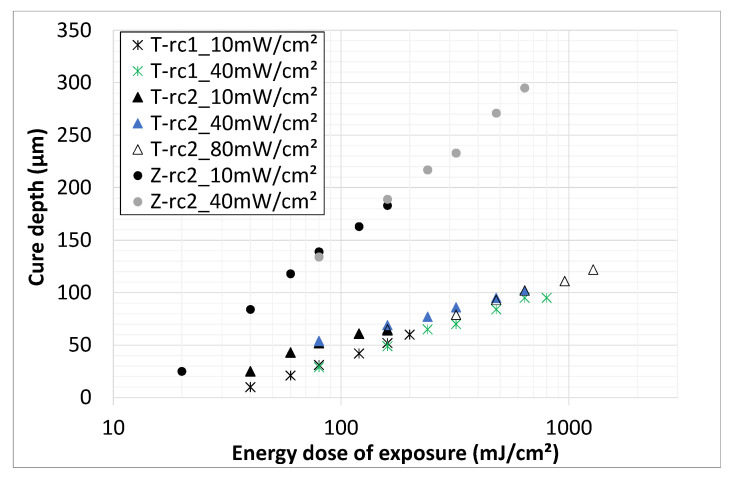
Cure depth (405 nm) vs energy dose for different intensities; titania (T) slurries based on rc1 and rc2; zirconia (Z) slurry based on rc2; particle content of 46 vol.%.

**Figure 8:**
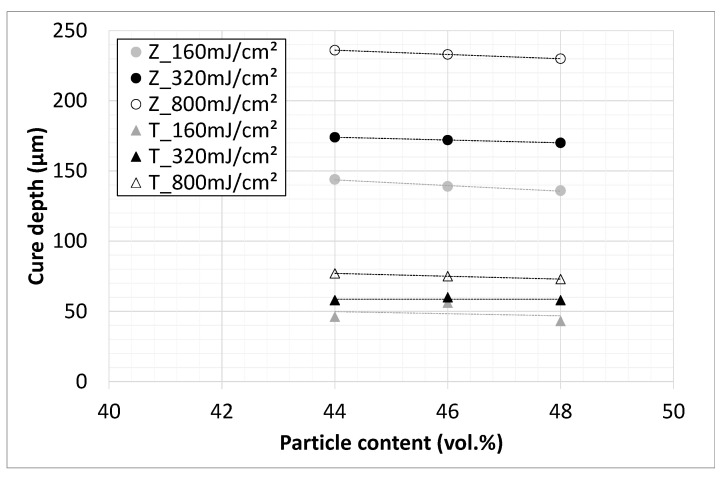
Cure depth (405 nm) depending on particle content; titania (triangles) and zirconia (circles) slurries based on rc2.

Based on the results, it seems that the intensity of the light source has no significant influence on the cure depth. The cure depth only depends on the energy dose of exposure (Figure 7). This means that, for a light source of higher intensity, a higher cure depth can be achieved with the same exposure time, which could be interesting in terms of economic aspects (saving time). The correlation between solid contents and cure depth at three different energy doses is shown in the next figure (Figure 8) for both materials.

Independently from the level of cure depth, which increases with exposure energy dose, a slow decrease with increasing solid content is evident in both materials. However, this decrease is small and can be neglected in the considered range of solid contents. It is assumed that the cure depth decreases only slightly with increasing solid contents (up to 51 vol.%).

**Figure 9:**
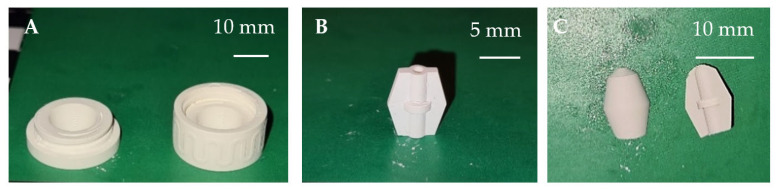
Printed “core–shell” in the green state, zirconia (**A**,**B**) and titania (**C**).

Based on the achieved results, printing parameters were derived for VPP for the different materials and slurries for printing test samples and validating the development. Starting with cubes, plates, bars and grid plates, first, parameter set-ups were tested, followed by printing more complex designs. After shrinkage characterization, the “core–shell” components introduced in Figure 1 were printed, and green parts are shown in Figure 9.

After printing and cleaning, the components were assembled, and the thermal co-processing was carried out.

### 3.2. Sintering

For both materials, it was possible to obtain components without visible defects. The material density determined by hydrostatic weighing was >6.01 g/cm³ (99.5% of theoretical density) for zirconia and approx. 4.04 g/cm³ for titania suboxide, although this density is strongly dependent on the final (mixed) phases of the structure. Pure, stable Ti_4_O_7_ or Ti_5_O_9_ phases could hardly be attained, they always appeared in a mixture of different non-stochiometric suboxides.

**Figure 10:**
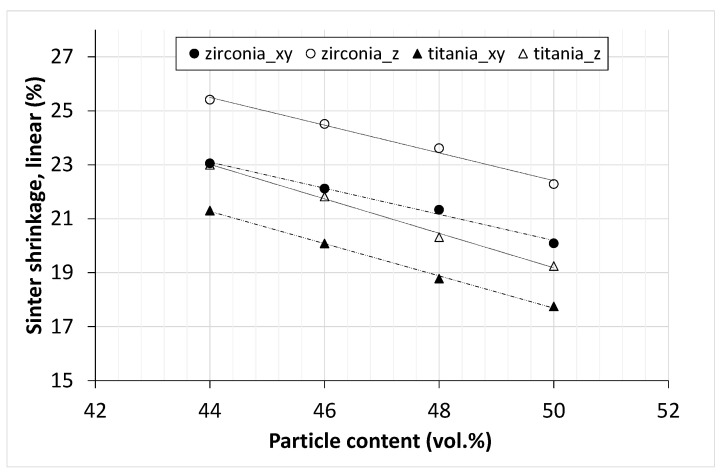
Sintering shrinkage in x-, y- and z-direction of zirconia (circles) and titania (triangles) depending on the solid content.

The shrinkage in all dimensions (x-, y-building plane and z-direction) of titania suboxide and zirconia components is presented in Figure 10, by plotting the sinter shrinkage as a function of the solids content of the printed components.

**Figure 11:**
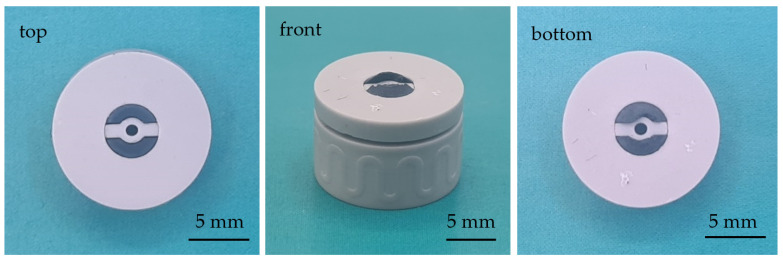
Co-sintered core–shell assembly; zirconia (gray) and electrically conductive titanium suboxide (blue-black).

Following the logical progression, the shrinkage for both materials decreases with increasing solid contents, whereby the trend seems linear within the considered range regardless of the direction. For both materials, the shrinkage is anisotropic and, in the z-direction, significantly higher (approx. 2–3%) compared to x- and y-direction, which is a typical phenomenon in lithographic ceramic printing. Furthermore, for comparable solid contents, the shrinkage of zirconia is noticeably higher compared to the titania shrinkage. This difference is also 2–3% but seems to increase with higher solid contents. One reason for the difference in shrinkage may be densification, which does not occur completely in titania and that leads to residual porosity. This fact is known and inherent, since, on the one hand, there is a phase transformation from anatase to rutile (at approx. 800–900 °C) and, on the other hand, there is a reduction with vapor formation that takes place during sintering. For experimental purposes, the combination of 43 vol.% zirconia and 47 vol.% titania was chosen, as this range was part of the characterizations. The core–shell assemblies (Figure 1) were produced with suspensions containing exactly this combination of solid contents. Figure 11 shows exemplarily a successfully sintered assembly of this component type.

### 3.3. Phases and Morphology of Sintered Structure

**Figure 12:**
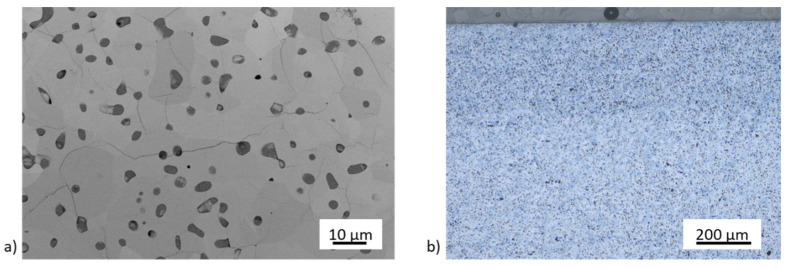
FESEM micrograph with microcracks (**a**) and an optical microscopic image showing a porous edge region (**b**) in the TiO_x_ substrate.

After the first heat treatments at a maximum temperature of 1450 °C, trans- and intercrystalline microcracks are visible in Figure 12, referring to a too high sintering temperature, which must be ≤1400 °C, even if a higher porosity must be accepted. Direct contact with the debinding and sintering nitrogen atmosphere leads to porous top edges (Figure 12b). No porous region is visible on the sample bottom.

**Figure 13:**
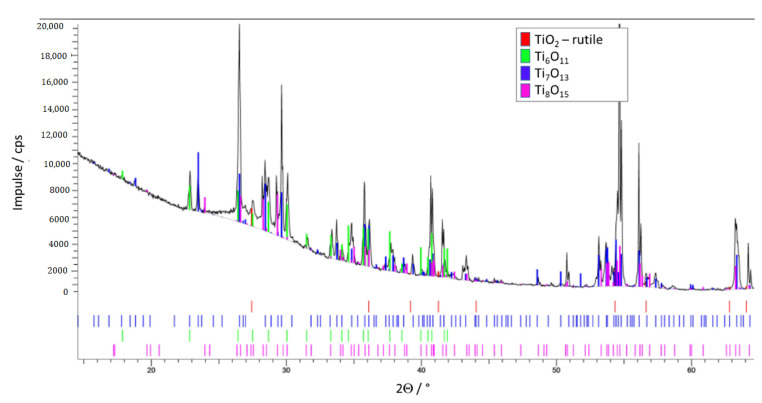
TiO_x_ phases at the surface of the test samples.

**Figure 14:**
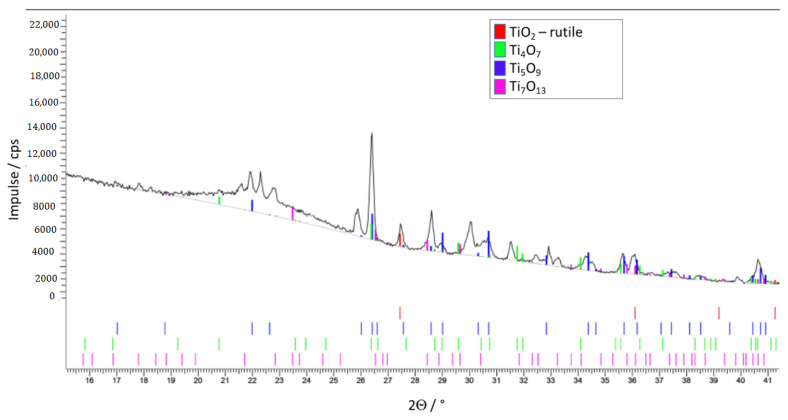
TiO_x_ phases in the core of the test samples; measured at a cross-section.

Due to the results of X-ray diffraction, the phases at the TiO_x_ surface exposed to the atmosphere are rutile, Ti_5_O_9_ and Ti_4_O_7_ (the last two with a high electrical conductivity), but also Ti_7_O_13_ (Figure 13). The same TiO_x_ phases can be seen along the cross-section of the substrate if it forms the outer part of the ZrO_2_–TiO_x_ composite. Phases at the cutting surface of the TiO_x_ substrate are shown in Figure 14. Close to the core of the composite are rutile and Ti_6_O_11_, Ti_8_O_15_ and Ti_7_O_13_, so-called Magnéli phases with worse electrical conductivity. The surface phases of TiO_x_ are different to the phases inside the bulk material. That is because of the incomplete and inhomogeneous TiO_2_ reduction during the heat treatment.

**Figure 15:**
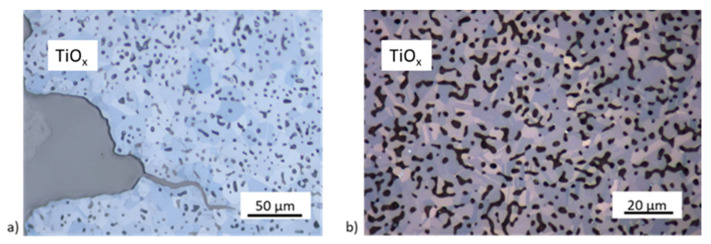
Twinning at a crack (**a**) and crack-free TiO_x_ substrate (**b**).

Twinning is present at the more porous edges and near one crack (Figure 15a), but not in the inner region of the sample. Figure 15b shows a homogenous sample texture and pore distribution with no significant differences between edges and inner texture after a heat treatment at 1400 °C. A few intergranular microcracks still exist. However, there are much fewer microcracks than in TiO_x_ samples sintered at 1450 °C and with a faster heating rate. This leads to a higher mechanical stability.

**Figure 16:**
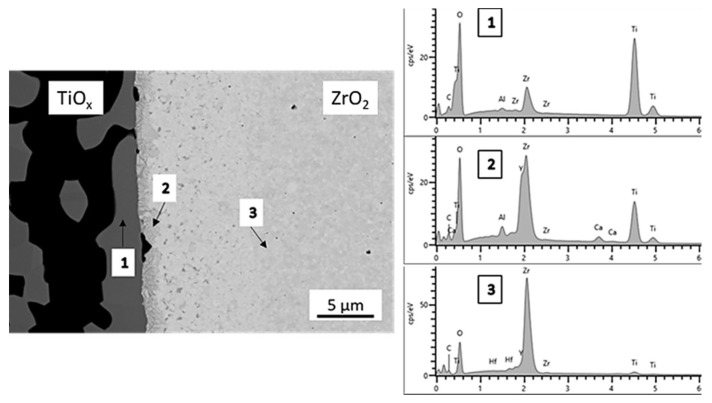
Interphase formation between ZrO_2_ and TiO_x_ in the sintered composite.

After sintering, in the composite made following the sintering regime given in Table 2, a homogenous sample texture of both materials was attained. A small gap is locally found because of different onsets of shrinkage and slightly different CTEs of both ceramic materials during cooling. Solid-state reactions at the interface between ZrO_2_ and TiO_x_ are possible (see Figure 16). The combination of these materials can lead to chemical bonding with a phase mixture of the available Zr-Ti-O elements. No additional form or force fitted bonding would be necessary with adjusted shrinkage.

### 3.4. Electrical Properties

**Figure 17:**
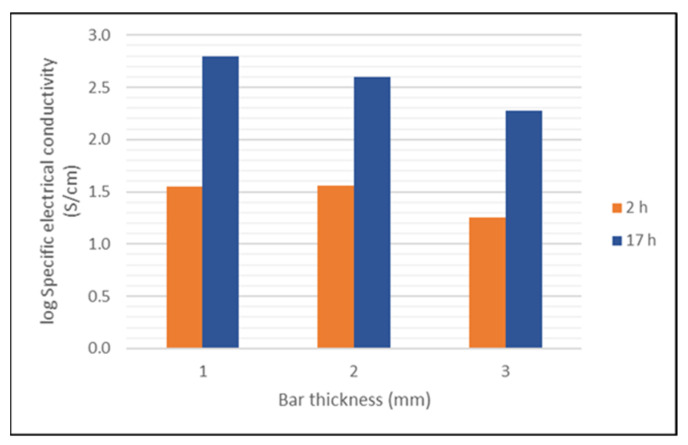
Specific electrical conductivity of titanium suboxide testing bars after sintering following the regime given in Table 2 with two different holding times at the transition temperature depending on the thickness of the bars.

After sintering in air and a subsequent thermal treatment at 1100 °C in hydrogen, a resistance of 3–6 Ω could be attained and, when sintering in pure hydrogen without a holding time, the resistance was 26–36 Ω. The results given in Figure 17 show that after sintering following the regime given in Table 2 and a holding time of 2 h at the transition temperature, electrically conductive parts can be attained, but after a holding time of 17 h, an electrical conductivity could be achieved for all three investigated bar thicknesses in the region of interest for practical applications.

## 4. Discussion

The results of our development work show that multi-functional ceramic components with the property combination of electrically conductive/electrically insulating cannot be attained via a vat photopolymerization process without further adjustments. The reason for this is the light-absorbing behavior of electrically conductive ceramic materials. In our case, we chose a detour in two-component VPP via the use of two translucent oxidic powders, zirconia and titania. Both powders are available and, moreover, titania, which is mainly used as white color pigment in paints, plastics, paper and others [31,32], is quite inexpensive. Zirconia and titania show a comparable CTE (ZrO_2_ = 10.8 × 10^−6^ K^−1^ [27], TiO_2_ = 7–8 × 10^−6^ K^−1^ [33,34,35] and can be co-sintered at 1400 °C. For attaining the desired properties of the material combination, the compound must be sintered under reducing gas atmosphere. A mixture of nitrogen/hydrogen of 95/5 was chosen for transforming the titania into a mixture of suboxides, among them the desired phases Ti_4_O_7_ and Ti_5_O_9_ with a high electrical conductivity. Meanwhile, the properties of the zirconia remain unchanged by the reducing gas atmosphere. The chosen sintering conditions resulted in a dense zirconia component which was still electrically insulating and a porous titanium suboxide component with electrical conductivity. Furthermore, the zirconia component remained mechanically stable, providing the compound with a remarkably high mechanical strength for application.

The VPP process provides some additional hurdles caused by the optical properties of titania. First, depending on the mineral phase of titania, either anatase or rutile, it shows an absorption edge for light with a wavelength shorter than approx. 400 nm [36]. With a curing wavelength of 405 nm, we are very close to this absorption edge and would need a much higher irradiation energy dose for a certain curing depth than with a curing wavelength of 465 nm. Second, titania has a very high refractive index (RI) in comparison to other ceramic powders which increases with decreasing light wavelength. According to the VPP process rules, the difference of the RI of the dispersed powder and the photocurable monomer matrix should be as small as possible. Unfortunately, the RI of the organic matrix ranged from 1.4 to 1.6, whereas rutile shows RI values between 2.8 and 3.3 depending on the wavelength. Thus, the titania powder particles cause a strong scattering of the curing light which is distributed to a high extent within the x–y-plane of the building layer rather than in the depth of the layer. This results in a lower resolution and a higher extinction of the curing light, and thus, in a lower curing depth. The curing depth in turn is the building speed-determining value in the VPP process. With a titania slurry with 40 vol.% solid content, a curing layer depth of 100 µm was attained after exposure to blue light of a 452–465 nm wavelength with 40 mW/cm^2^ only after 20 s (800 mJ/cm^2^). After a 20 s exposure of the same slurry with light of a 405 nm wavelength with the same energy dose, the curing depths dropped down to only 70 µm, because the systems moved closer to the absorption edge of titania and, moreover, the RI increased further. Compared with zirconia, the curing speed can be more than doubled. Nevertheless, titania and zirconia powders are suited for the VPP process and the multi-material process variant of VPP would allow for making complex shaped parts which can be co-sintered afterwards for a transformation of the titania into its electrically conductive suboxides.

During the co-firing process in a reducing atmosphere, the titania loses lattice oxygen and non-stochiometric suboxides are formed and, among them, the compositions Ti_4_O_7_ and Ti_5_O_9_ are the most desired due to their high electrical conductivity [37]. The desired pure phase can hardly be attained by gas atmosphere control during sintering [38]. Moreover, the reduction process of the titania depends on the holding time at the transition temperature, the particle size of the titania powder and the thickness of the component and further influences. As shown in Figure 17, after 17 h of sintering in a forming gas atmosphere, a suboxide mixture in the testing bars was attained, showing a specific electrical conductivity which could be compared to values reported by other authors [36,37]. The decrease in the electrical conductivity with increasing thickness of the testing bars reflects the influence of the reaction kinetics on the phase transition from TiO_2_ into titanium suboxides until reaching an optimal composition with the desired phases. The oxygen loss of the titania is much faster close to the surface of a component than within the bulk material. During this thermal treatment, the zirconia component remains largely uninfluenced by the sintering atmosphere.

It is exactly this that makes the material combination of titanium suboxides and zirconia so promising for electrical or thermoelectrical applications. While the suboxides fulfill their task as electrically conductive phases, the zirconia provides the electrical insulation coupled with high mechanical strength. Surprisingly, no scientific papers on this material combination have come to our attention to date. Two-component VPP could now offer a suitable manufacturing technique for producing novel multi-functional components with the highest complexity.

In the next step of our collaborative project, two-component test samples with a solids content based on the sintering shrinkage study, described in Section 2.5, shall be printed with the multi-CAMP device of KIMS.

## 5. Conclusions

The results of this work will be used for enabling the multi-CAMP machine based on the vat photopolymerization technology to produce ceramic components consisting of two different materials with the property combination of electrical conductivity/electrical insulation in the near future. The to date impossible material combination for this additive manufacturing method has been made feasible by using the fact that white, translucent titania powder, which is suited for building parts by the VPP process, can be transformed into titania suboxides with high electrical conductivity, but a dark color during sintering, in a reducing gas atmosphere. Titania and zirconia show comparable sintering behavior, i.e., both ceramics can be densified under comparable sintering conditions. Both components attain their final properties during co-sintering, i.e., while the titania is reduced into titania suboxides with high electrical conductivity, the zirconia part is densified for high mechanical strength, but remains electrically insulating.

The described material combination with its interesting properties can be made by means of conventional shaping routes in a more direct way just by combining green components of zirconia and titanium suboxide powders and co-sintering in a reducing gas atmosphere, but when taking advantage of the opportunities of the VPP process for building components with a to date impossible geometrical complexity, the above-described detour via the combination of two green parts made of translucent initial powders is indispensable.

## Figures and Tables

**Table 1:** Monomers and compositions used for zirconia and titania slurries.

Formulation	Component A	Component B	Component C	Mixture Ratio A:B:C
rc1	aliphatic urethane acrylate (Laromer UA9089; BASF)	isobornylmeth-acrylate (Photomer 2012; IGM resin)	1.6-hexanediol diacrylate (Laromer HDDA, BASF)	2:2:6
rc2	4-hydroxybutyl-acrylate (4-HBA; TCI Deutschland GmbH)	polyether acrylate (Ebecryl40, Allnex GmbH)	3-methyl-1.5 pentanediol diacrylate (Photomer 4071; IGM resin)	5:2:3

**Table 2:** Sintering regime of TiO_x_–ZrO_2_ composite components with reduction of TiO_2_ to TiO_x_.

Heating Rate	Target Temperature	Atmosphere	Holding Time	Atmosphere after Holding Time
5 K/min	620 °C	N_2_/H_2_ 95/5 flushing	-	-
1 K/min	1200 °C	N_2_/H_2_ 95/5 flushing	1020 min	N_2_/H_2_ 95/5 flushing
-	1200 °C	N_2_/H_2_ 95/5 flushing	10 min	Vacuum
3 K/min	1400 °C	N_2_ standing	120 min	N_2_ standing
3 K/min	1200 °C	N_2_ standing	10 min	N_2_/H_2_ 95/5
3 K/min	Room temperature	N_2_/H_2_ 95/5 standing	-	-

## Data Availability

The data are not publicly available due to data confidentiality resulting from the requirements of the accreditation.
